# Fulminate Hepatic Failure in a 5 Year Old Female after Inappropriate Acetaminophen Treatment

**DOI:** 10.3889/oamjms.2015.080

**Published:** 2015-09-08

**Authors:** Irena Kasmi, Sashenka Sallabanda, Gentian Kasmi

**Affiliations:** 1 *Pediatric Department University Hospital Center, Tirana, Albania*; 2 *Laboratory Department University Hospital Center, Tirana, Albania*

**Keywords:** acetaminophen, toxicity, liver failure, NAC, pancreatitis

## Abstract

**BACKGROUND:**

Acetaminophen is a drug widely used in children because of its safety and efficacy. Although the risk of its toxicity is lower in children such reactions occur in pediatric patients from intentional overdoses and less frequently attributable to unintended inappropriate dosing. The aim of reporting this case is to attract the attention to the risk of the acetaminophen toxicity when administered in high doses.

**CASE PRESENTATION:**

We report here a 5 year old girl who developed fulminate liver failure with renal impairment and acute pancreatitis, as a result of acetaminophen toxicity caused from unintentional repeated supratherapeutic ingestion, with a total administered dose of 4800 mg in three consecutive days, 1600 mg/day, approximately 90 mg/kg/day. The blood level of acetaminophen after 10 hours of the last administered dose was 32 mg/l. The patient presented with high fever, jaundice, lethargic, agitating with abdominal pain accompanied by encephalopathy. The liver function test revealed with high level of alanine aminotransferase 5794 UI/l and aspartate aminotransferase 6000 UI/l. Early initiation of oral N-acetylcysteine (NAC) after biochemical evidence of liver toxicity was beneficial with rapid improvement of liver enzymes, hepatic function and encephalopathy. During the course of the illness the child developed acute pancreatitis with hyperamylasemia 255 UI/L and hyperlypasemia 514 UI/L. Patient totally recovered within 29 days.

**CONCLUSION:**

Healthcare providers should considered probable acetaminophen toxicity in any child who has received the drug and presented with liver failure. When there is a high index of suspicion of acetaminophen toxicity NAC should be initiated and continued until there are no signs of hepatic dysfunction.

## Introduction

Acetaminophen is the most widely used analgesic and antipyretic drug in infants and children. The safety of acetaminophen is well established especially in comparison with aspirin. Therefore it is used over the counter and on prescription. The recommended maximum therapeutic dosage in symptomatic fever and analgesia in children is 50 to 75 mg/kg/d [1].

Acetaminophen poisoning remains one of the more common drugs taken in overdose with potentially fatal consequences. A single acute ingestion of greater than 7.5 g in an adult or 150 mg/kg in children has been considered potentially toxic [2]. In the United Kingdom, nearly 50% of drug poisoning was due to it and the mortality was about 100 to 200 victims per year. Reviewing database in the Toxic Exposure Surveillance System of the American Association of Poisson Control in 2004, 19 590 cases were under the age of six [3].

In general the risk of developing toxic reactions appears to be lower in children than in adults [4]. The most common organ affected by acetaminophen intoxication is the liver when administered in supratherapeutic doses or repeated therapeutic doses can also be toxic [4, 5]. Because the symptoms of acetaminophen intoxication are nonspecific the diagnosis and the treatment are more likely to be delayed in such cases. The health care providers should consider acetaminophen toxicity as a probable diagnosis in any child who has received the drug and has sign of hepatic failure. Early initiation of NAC is indicated whenever the acetaminophen is considered, as the delays in the treatment are associated with a worse outcome [4].

The aim of this report is to present fulminate hepatic failure in a 5 year old female after inappropriate acetaminophen treatment.

## Case Report

### Day 1

The 5 year old female child enjoyed good health in the past until three days before the admission when she began to have high fever, vomiting and diarrhea. The child was treated by the mother with different formulation of acetaminophen as suppositories 250 mg and tablets 500 mg every 3 hours for three consecutive days. The used dose of acetaminophen was calculated by the physician at the admission time to Pediatric Intensive Care Unit of UHC, being approximately 90 mg/kg/24h for the last 72 hours. No other concomitant medication was administered.

At the admission the child presented with high fever, vomiting, confusion, drowsiness alternated with agitation especially to external stimuli. On examination, absent neck stiffness, Babinski response was negative, pupils were slightly midriatic, corneal reflex was present, sensible only to pain according to AVPU evaluation and was classified as grade II to III of encephalopathy, dehydrated and icteric. The arterial pressure was 90/50 mmHg, heart rate was 160/min, the respiratory rate was 45/min the temperature was 39°C, the pulsoximeter 97% saturation in room air. Abdominal palpation revealed liver enlargement 2-3 cm under the costal arch, and a soft palpable abdomen. The bedside blood sugar was 45 mg/dl, ALT 5794 UI/L, AST 6000 UI/L, Total Bilirubine 3.5 mg/dl, INR 3.86, blood urea 80 mg/dl, blood creatinine 1.3 mg/dl, 8GT 60 UI/l, Na+ 111 mmol/l, K+3.5 mmol/l. The blood albumin level 3 g/dl, Red blood cells 4.4*10^12^/L, Hemoglobine 12.3 g/dl, Platelets 332*10^9^/L, White blood cell 25*10^9^/L C reactive Protein 70 mg/dl. A careful disease history was taken and acetaminophen poisoning was strongly suspected. The blood acetaminophen obtained 10 hours after the last administered dose, was sent abroad having the positive result 32.9 mg/l. The viral serologic profile includes HIV, HEV, HAV, HCV, HBV, Coksaxie Virus, influenza A, B, Adeno Virus, was negative. The chest radiograph was normal. Blood culture failed to isolate any microorganism. NAC was immediately given by nasogastric tube as the only drug formulation the hospital could provide according to the standard oral guideline with 140 mg/kg loading dose followed by 70 mg/kg every 4 hours for a total of 18 doses over 72 hours. Oral feeding was stopped and the hydration was maintained by electrolytic solution.

**Table 1:** Serial Laboratory Investigations in our patient

Investigation	Day 1	Day 2	Day 3	Day 5
Hb (g/dl)	12.3		9.5	
Total white counts/L	25*10^9^		22*10^9^	
Platelets/L	332*10^9^		444*10^9^	
Albumin (g/dl)	3	3.2	4.4	4.4
Bilirubin (mg/dl)	3.5	2.5	1.4	0.5
ALT (U/L)	5794	3250	570	58
AST (U/L)	6000	2900	490	95
GGT (U/L)	60			
Amylase (U/L)			255	43
Lypase (U/L)			514	
Creatinine (mg/dl)	1.3	1.1	0.98	0.45
Urea (mg/dl)	80	50	43	10.3
Blood sugar (mg/dl)	45	90	340	110
Acetaminophen (mg/L)	32.9			
INR	3.86	3.14	2.13	
C reactive protein (mg/dl)	70			100

Hb: haemoglobin; ALT: alanine aminotransferase; AST: aspartate aminotransferase; GGT: gamma glutamyltranspeptidase; INR: international normalised ratio.

### Day 2-5

After administration of 18 oral recommended NAC doses of hepatic failure treatment protocol, the renal indicators as blood creatinine, blood urea and the liver enzymes were improved but without INR. The coagulopathy aggravated with clinical manifestations of melena and hematemesis despite the therapy with fresh frozen plasma, Vitamin K, Human Albumin, Acid tranexamic, Lactulose. Abdomen became painful and the blood sugar 340 mg/dl, hypernatremia 160 meq/l, hypokalemia 2.7 meq/l. Acute pancreatitis was suspected and confirmed by hyperamylasaemia 255 UI/l, hyperlypasaemia 514 UI/l, and ultrasound examination which revealed a swollen pancreas and minimal peritoneal liquid at Douglas space. Accordingly the therapy was modified to parenteral hydration, fluid and electrolytes correction and reduces enteral stimulation. A further 24 hours of NCA treatment was added until the INR and pancreatic enzyme decline.

### Day 7

As the patient condition improved in terms of hepatic failure and clinical acute pancreatitis, the fever remained high with chills and high spikes. The patient developed left leg pain with thrombophlebitis and coccygofemoral arthritis.

The repeated blood culture finally isolated Klebsiella pneumoniae despite the extendum spectrum antibiotics (Cefuroxime 150 mg/kg/day and gentamicin 5 mg/kg/day) and the therapy was adopted according to antibiotic susceptibility (Piperacillin-Tazobactam and Amikacin) supported with Pentaglobine. This situation required also the use of anticoagulant (Fraxiparin 2850 UI s/c) and Ibuprofen. Despite the long progress of the disease the child recovered totally within 29 days with a complete resolution of all clinical and laboratory abnormalities. The liver function tests remained in normal range during the following up of 1st, 3rd, 6th and 12th month.

## Discussion

In Albania acetaminophen toxicity in children is uncommon. Many parents use this medication without consulting doctors. The aim of reporting this case was to attract the attention to the risk of the acetaminophen toxicity when administered in high doses.

When it happens acetaminophen-induced hepatotoxicity remains a serious condition in pediatric practice [6]. Cases of acetaminophen toxicity induced by multiple therapeutic or supratherapeutic doses have been reported [7]. Recent reviews identified several factors associated with acetaminophen hepatotoxicity and other extra-hepatic manifestations in children including: age less than 10 years, delays in onset of symptoms after a potentially toxic ingestion, delay in initiation of NAC treatment, unintentional multiple overdosing, ingestion of acetaminophen with other hepatotoxic drugs and the use of adult rather than pediatric preparations [4].

In the reported case the hepatic failure was caused by using unintentionally repeated excessive dosing of acetaminophen as pediatric preparation in suppositories 250 mg combined with adults preparations in tablet of 500 mg, every 3 hours for three consecutive days. The calculated dose of the used acetaminophen in our case was approximately 90 mg/kg/24h for more than 72 hours which almost compiles with the recent expert panel’s guideline in management of acetaminophen RSTI (repeated supratherapeutic ingestion). According to this panel’s guideline, doses 100 mg/kg or more per 24 hours for 72 hours or more, are doses of concern for liver injury in children younger than 6 years [8]. Lower doses in sick children younger than 2 years in presence of risk factors such as febrile illness, prolonged starvation, diarrhea, repeated vomiting and poor fluid intake more than 24 h may suffice to cause hepatotoxicity [9]. Acetaminophen is metabolized in the liver via 3 main pathways: sulfation, glucuronidation and oxidation. The first 2 ways are quantitatively more important, but oxidative pathway is implicated in toxicity. The sulfate pathway may become depleted during repeated multiple doses of actaminophen which result in hepatotoxicity because the drug is oxidized by CYP (CYP2 E1, 3A4, and 1A2) to the toxic metabolite (N-acetyl-p-benzoquinone imine{NAPQI}) that is detoxified by glutathione. In overdose cases glutathione declines and the toxic NAPQI fraction produces hepatic necrosis [10]. Many reported cases of severe hepatoxicity in children have been attributed to cumulative toxicity from repeated doses rather than acute intoxication from a single massive overdose [4]. In our case the serum level of acetaminophen was performed abroad and the result came late while the diagnosis was concluded based on clinical presentation and the medical history. In fact the assessment of serum acetaminophen levels is useful in the management of the overdose when the time of ingestion is known with certainty. In such cases the Rumack-Mathews nomogram is a valuable tool to assess the risk of hepatotoxicity. However, if the overdose is staggered over a long period, serum acetominophen tests are impossible to be interpreted by this nomogram [2]. The symptoms of acetaminophen intoxication are nonspecific, making diagnosis and treatment of unintentional acetaminophen intoxication more likely to be delayed [11], as it happened in our case. While the acetaminophen poisoning has primarily been associated with hepatoxicity, a range of less common extrahepatic manifestations has also been described. Among these, nephropathy has been recognized as a clinically important complication and considerable prognostic implications [12]. In the current case the renal involvement was present at the admission time and renal indicators improved rapidly along the therapy. Pancreatitis, cardiotoxicity and hematotoxicity are the more unusual complication of acetaminophen poisoning. During the disease course, the clinical and laboratory diagnosis of pancreatitis was made based on hyperlypasaemia, hyperamylasaemia level, hyperglycemia and changes on abdomen ultasonography. Acute pancreatitis has been described between 6 and 41% of patients with fulminant hepatic failure FHF. Although hyperamylasemia is a common finding in patients with acetaminophen poisoning, clinical signs of pancreatitis are rare only a few cases of acetaminophen induced pancreatitis have been reported. In one study performed in Denmark hyperamylasaemia occurred in 80% acetaminophen induced-FHF while only 13 % was diagnosed with clinical pancreatitis and only two cases with actetaminophen – induced pancreatitis without liver and kidney involvement [12]. In the current case diagnosis of pancreatitis was suspected and confirmed by the third day. N-acetylcysteine NAC is an effective antidote for acetaminophen poisoning. When administered early dramatically reduces the incidence of hepatotoxicity and the FHF. NAC provides cysteine for the replenishment and maintenance of hepatic glutathione stores enhances the sulfation pathway of elimination or directly reduces NAPQI back to acetaminophen. Patients with severe hepatic injury also benefit from NAC. The mechanism here is not the detoxification of NAPQI but the enhanced recovery through improvement of hepatic perfusion and oxygen delivery and extraction, mitochondrial energy production, scavenge of reactive oxygen and nitrogen. NAC is available both orally and intravenously. Both regimes are effective for the treatment of an acute acetaminophen overdose. Whatever the route of administration following an acute acetaminophen ingestion, the treatment should not be delayed beyond 8 hours. Conversely in patients with hepatotoxicity NAC should be continued beyond the usual course of therapy in any patients with persistent signs of liver injury [2]. In the current case the effectiveness was evident with the first 18 oral doses in terms of dramatic decline in liver enzymes while the INR improvement and pancreatic enzymes decline came after an additional 24 hours of NAC administration. We consider the decision to continue the oral NAC beyond the usual course reasonable.

In conclusion, although acetaminophen is considered a safe drug in children it has the potential for hepatoxicity not only in intentional overdoses but even with unintended inappropriate doses. Healthcare providers should considered probable acetaminophen toxicity in any child who has received the drug and presented with liver failure. When there is a high index of suspicion of acetaminophen toxicity NAC should be initiated and continued until there are no signs of hepatic dysfunction.
